# Impact of chronic immobilization stress on parameters of colonic homeostasis in BALB/c mice

**DOI:** 10.3892/mmr.2019.10437

**Published:** 2019-06-27

**Authors:** Nancy Machorro-Rojas, Teresita Sainz-Espuñes, Marycarmen Godínez-Victoria, Jorge Ismael Castañeda-SÁnchez, Rafael Campos-Rodríguez, Judith Pacheco-Yepez, Maria Elisa Drago-Serrano

**Affiliations:** 1 Departamento de Sistemas Biológicos, Universidad Autónoma Metropolitana, Unidad Xochimilco, Mexico City 04960, Mexico; 2 Sección de Estudios de Posgrado e Investigación, Escuela Superior de Medicina, Instituto Politécnico Nacional, Mexico City 11340, Mexico

**Keywords:** board immobilization stress, goblet cells, tight junction proteins, fecal lactoferrin, bacterial overgrowth, colon

## Abstract

The intestinal epithelium is a monolayer of cells arranged side-by-side and connected by tight junction (TJ) proteins expressed at the apical extreme of the paracellular membrane. This layer prevents stress-induced inflammatory responses, thus helping to maintain gut barrier function and gut homeostasis. The aim of the present study was to evaluate the effects of chronic immobilization stress on the colonic expression of various parameters of homeostasis. A total of two groups of female BALB/c mice (n=6) were included: A stressed group (short-term immobilization for 2 h/day for 4 consecutive days) and an unstressed (control) group. Colon samples were obtained to detect neutrophils and goblet cells by optical microscopy, TJ protein expression (occludin, and claudin −2, −4, −7, −12 and −15) by western blotting, mRNA levels of TJ genes and proinflammatory cytokines [tumor necrosis factor (TNF)-α, interleukin (IL)-1β, −6 and −8] by reverse transcription-quantitative PCR, fecal lactoferrin by ELISA and the number of colony-forming units of aerobic bacteria. Compared with goblet cells in control mice, goblet cells were enlarged and reduced in number in stressed mice, whereas neutrophil cellularity was unaltered. Stressed mice exhibited reduced mRNA expression for all evaluated TJ mRNAs, with the exception of claudin-7, which was upregulated. Protein levels of occludin and all claudins (with the exception of claudin-12) were decreased in stressed mice. Fecal lactoferrin, proinflammatory cytokine mRNA levels and aerobic bacterial counts were all increased in the stressed group. These results indicated that immobilization stress induced proinflammatory and potential remodeling effects in the colon by decreasing TJ protein expression. The present study may be a useful reference for therapies aiming to regulate the effects of stress on intestinal inflammatory dysfunction.

## Introduction

The effective functioning of the intestinal barrier is important for the maintenance of homeostasis in this organ, as the intestinal epithelium acts as the interface between the outer and inner microenvironments (1). The intestinal epithelium is a polarized monolayer that functions as a selective filter, permitting the transport of luminal nutrients and preventing the passage of harmful agents towards the inner milieu (1).

Monolayer components, such as absorptive enterocytes, are connected adjacently at the apical side of the paracellular membrane by tight junctions (TJs), which have a key role in the regulation of intestinal permeability (2). TJs are composed of integral transmembrane proteins, including claudins and occludin (a member of the TJ-associated MARVEL protein family), and junctional adhesion molecules (JAM)-A, -B and -C (2). In the intracellular milieu, TJs interact with the perijunctional actomyosin ring of cytoskeleton filaments via the periplasmic scaffolding protein zonula occludens-1 (ZO-1) (2). At the extracellular level, claudins serve as barrier-enhancers by forming ‘kisses’ that seal the paracellular space, or enable a paracellular flux of solutes via pore-forming or leaky pathways (3). By contrast, occludin is involved in the regulation of the flux pathways (3). In the mouse colon, claudins of the cation-pore (claudin-7, −12 and −15) or cation-barrier (claudin-8) type increase electrolyte absorption or decrease permeability, respectively (4–6). In mice, claudin-8 is more strongly expressed than claudin-12 and −15 in the colon, whereas claudin-7 and occludin are evenly distributed in the intestinal tract (4–6).

The mucus layer produced by goblet cells is an extracellular component that serves an important role in intestinal barrier function. Mucin 2, which blocks the direct contact of microbiota with the epithelial surface to prevent an inflammatory reaction, is located within the mucus layer (7). The colon comprises an outer loose mucus layer rich in microbiota, and an inner layer devoid of bacteria and tightly attached to the epithelium (7).

Chronic stress can alter the function of the colonic barrier, leading to increased permeability, bacterial overgrowth, infiltration of neutrophils and mucus depletion (8–11). The deleterious effects of chronic stress on barrier function result in part from the activation of the hypothalamus-pituitary-adrenal (HPA) axis and the concomitant release of corticotropin releasing factor, which induces the activation and degranulation of colonic mast cells (11–15). Compounds released by mast cells induce a wide array of effects on neuroendocrine and immune pathways that regulate colonic permeability and replenish the mucus (11–15). Stress hormones released via HPA activation, such as adrenal corticosteroids, are also involved in the control of colonic permeability (16).

Assays in which mice or rats underwent water-avoidance stress or crowding stress have been used to investigate the effects of chronic stress on the protein and mRNA expression of certain TJ components in the colon (such as ZO-1, JAM-A, occludin and claudin-1, −2, −5 and −8), mucus properties and the inflammatory response (11,16–19).

At present, the impact of chronic immobilization stress on TJ proteins and other biomarkers involved in gut barrier function are yet to be reported. Therefore, the present study aimed to conduct an overall analysis of the effects of chronic immobilization stress on epithelial components (goblet cells, TJ proteins) and nonepithelial biomarkers (neutrophils, fecal lactoferrin, proinflammatory cytokines, aerobic bacteria) involved in colonic homeostasis.

## Materials and methods

### 

#### Animals

A total of 12 6-week-old female BALB/c mice (Unidad de Production y Experimentacion de Animales de Laboratorio, Universidad Autonoma Metropolitana Unidad Xochimilco) were housed in two groups (n=6 in each group) under a 12-h light/dark cycle (the light phase begins/ends at 7:00 a.m./7:00 p.m.), in a room at 20°C, with a relative humidity of 55%. The mice were allowed free access to food and water (Laboratory Rodent Diet 5001; LabDiet) *ad libitum*. Mice were housed for 2 weeks prior to the initiation of the stress protocol to adapt to housing conditions. Animal manipulations were always performed by the same trained handler between 8:00-11:00 a.m. to reduce the influence of the circadian cycle on fluctuations in the levels of corticosterone and adrenocorticotropic hormones. The animal experiments (approval no. 176) were approved by The Comite Interno para el Uso y Cuidado de Animales de Laboratorio, Universidad Autonoma Metropolitana, Unidad Xochimilco. Animals were maintained and handled according to the Mexican federal regulations for animal experimentation and care (NOM-062-ZOO-1999; Ministry of Agriculture, Mexico City, Mexico) (20).

#### Stress protocol

At 8 weeks of age, mice were subjected to restraint stress or control conditions. During the short-term stress protocol, a group of mice (n=6) was immobilized on a board, as previously described (21). In addition, a control (untreated) group (n=6) was included. For immobilization, mice were placed in a prone position, and the four limbs were gently stretched and attached with adhesive tape to an expanded polystyrene board that was covered with plastic film for easy cleaning. Immobilization was first applied to the fore feet, then the hind feet and finally the tail. Very low-adhesion tape was fastened directly to the skin of the animals on top of which high-adhesion tape was placed, thus minimizing pain during tape removal. Adhesive tape was placed on the dorsum of the fore feet, the foot pads of hind feet, and the middle part of the tail. Curve strips produced from paperboard-adhesive tape reels were placed upon the adhesive tape as chewers for mice to prevent self-inflicted injuries on the fore leg skin. During the assay, the head of each mouse was allowed to move freely, whereas the twisting of limbs and tearing of whiskers were reduced. Following immobilization for 2 h, the adhesive tape was carefully removed in the following order: Tail, hind feet and fore feet. This protocol was repeated daily for 4 days, beginning at the same time every day.

#### Collection of biological samples

Upon completion of the stress protocol, fecal pellets were collected in microcentrifuge (2 ml) tubes. All animals were subsequently sacrificed via exposure to isoflurane and exsanguination by cardiac puncture. The colon was dissected and flushed with sterile PBS (pH 7.2) to remove all luminal fecal content. Then, 1-cm samples of the colon were cut for histological examination by optical microscopy. Additional 1-cm colonic segments were collected in sterile pre-weighed microcentrifuge tubes (2 ml) containing 500 µl thioglycolate broth for bacterial counts. The colonic mucosa was extracted using a glass microscope slide and stored at −70°C for western blot and or reverse transcription-quantitative PCR (RT-qPCR) analyses.

#### Neutral and acid mucin staining procedure

Colon tissues were fixed with 4% paraformaldehyde for 30 min at 37°C and embedded in paraffin. Tissues were cut into 7-µm-thick sections. Neutral or acid mucins in colonic samples were detected by staining with periodic acid-Schiff (PAS) or alcian blue (AB), respectively. Following deparaffinization with xylene for 30 min at 60°C and rehydration in a graded alcohol series, samples were incubated in 0.5% periodic acid for 15 min at room temperature, washed with water and incubated with Schiff's reagent for 30 min at room temperature. The samples were then washed in warm water. For AB staining, samples were incubated in 3% acetic acid for 3 min at room temperature and then incubated in AB solution (1% in 3% acetic acid) for 15 min at room temperature. The slides were then washed in water, as previously described (22). The morphology of PAS- and AB-positive cells was visualized by light microscopy. Stained cells were counted in five randomly-selected fields of view (area of each field of view, 0.05 mm^2^) from each mouse (magnification, ×40; ECLIPSE E 600; Nikon Corporation). The number and size of cells were analyzed using Image-Pro Plus 5.1 software (Media Cybernetics, Inc.).

#### Determination of fecal lactoferrin

The determination of fecal lactoferrin was conducted using a protocol based on an indirect ELISA with certain modifications (23). The weight of two fecal samples was measured from the difference between the weight of the tubes before and after collection. Then, feces were fully homogenized in 500 µl of collection buffer [PBS with protease inhibitor cocktail (c*O*mplete Mini; cat. no. 11836153001; Roche Diagnostics) and 0.1% sodium deoxycholate (cat. no. D5670; Sigma-Aldrich; Merck KGaA)]. Fecal suspensions were centrifuged at 4°C, for 10 min at 10,000 × g, and supernatants were collected and stored at −70°C. Total protein content was quantified using the Bradford method (cat. no. 500-0006; Bio-Rad Protein Assay, Bio-Rad Laboratories, Inc.). Fecal extracts were analyzed in ≥3 replicates. The total volume of all reactions was 100-µl. Microtiter plates (96-well; cat. no. 3590, Costar; Corning, Inc.) were coated with fecal extracts (total protein content, 50 µg/ml) in carbonate-bicarbonate buffer (pH 9.6) and incubated overnight at 4°C. The plates were washed three times with 200 µl PBS with 0.05 % Tween 20. The samples were blocked with 0.5% BSA (cat. no. A2934; Sigma-Aldrich) (24) in carbonate-bicarbonate buffer (pH 9.6) for 2 h at 37°C. After washing, the samples were incubated with rabbit anti-human lactoferrin (cat. no. L3262; Sigma-Aldrich) in 0.5% BSA/PBST and incubated overnight at 4°C. Plates were washed and then incubated for 1 h at 37°C with horseradish peroxidase (HRP)-conjugated goat anti-rabbit IgG (cat. no. ab97080; Abcam; 1,5,000) in 0.5% BSA/PBST. The substrate mixture [30% hydrogen peroxide (10 µl) and 10 mg *o*-phenylenediamine dissolved in 0.05 M citric-phosphate buffer (50 ml) at pH 5.0] was subsequently added prior to incubation at room temperature for 20 min. The enzymatic reaction was stopped with 2.5 M sulfuric acid, and the absorbance was detected at λ=492 nm using a microplate reader. A standard curve of human lactoferrin was generated to quantify the lactoferrin concentration and was divided by the fecal sample weight to calculate the lactoferrin concentration in ng/g (23).

#### mRNA expression assays

RNA was isolated from colonic mucosa using TRI Reagent^®^ (cat. no. TR 118, Molecular Research Center, Inc.). Total RNA (0.2 µg) was used as template. The RNA was mixed with 1 µl Oligo dT 15 primer (cat. no. C1101; Promega Corporation) and sterile injectable water in a total volume of 25 µl. The reaction mixture was heated at 70°C for 5 min and immediately cooled on ice. Subsequently, the mixture was incubated with 5 µl M-MLV 5X reaction buffer, 1.0 µl M-MLV reverse transcripatse (both from Promega Corporation), 1.5 µl dNTPs mix (Promega Corporation) and sterile water in a total volume of 25 µl. The reaction mixture was heated at 42°C for 1 h using a thermocycler. cDNA was quantified using a spectrophotometer (Nanodrop 2000, Thermo Fisher Scientific, Inc.), and purity and integrity were evaluated by electrophoresis on a 1.5% agarose gel. The qPCR reaction was performed as follows: 20 µl Taq DNA polymerase master mix 1.1X (cat. no. A120301; Ampliqon, Inc.), 1 µl forward and 1 µl reverse primers (10 µM), 1 µl cDNA (100 ng), 1 µl EvaGreen Dye (cat. no. 31000; Biotium, Inc.) and sterile water in a total volume of 25 µl. qPCR was performed using a Gene 6000 Rotor (Qiagen GmbH) as follows: Initial denaturation at 95°C for 10 min, followed by 40 cycles of 95°C for 10 sec, 60°C for 15 sec, and 72°C for 20 sec. The relative gene expression values of TJ proteins (occludin, claudin-2, −4, −7, −12 and −15) and proinflammatory cytokines (TNF-α, IL-1β, −6 and −8) were determined using the 2^−ΔΔCq^ method (25) and normalized to the constitutive gene β-actin. Specific oligonucleotide primers for TJ proteins, cytokines and β-actin (Table I) were designed using the sequences provided in the database of genes at the National Center for Biotechnology Information and Primer3 version 0.4.0 software (26). The data were analyzed using RegLinPCR version 2015.3 software (http://www.hartfaalcentrum.nl).

#### Western blot assay

Extracts from colonic mucosa were homogenized in 500 µl lysis buffer (0.1 M Tris-HCl pH 7.5 containing 2% SDS, 10% glycerol, 5% 2-mercaptoethanol with a protease inhibitor cocktail (c*O*mplete Mini; cat. no. 11836153001; Roche Diagnostics) as previously described (27). Total protein lysates were quantified using Bradford assay (Bio-Rad Laboratories, Inc.). In total, 50 µg protein extracts were mixed with NuPAGE LDS sample buffer (4X; cat. no. NP0007; Invitrogen; Thermo Fisher Scientific, Inc.), NuPAGE™ Sample Reducing Agent (10X; cat. no. NP0009; Invitrogen; Thermo Fisher Scientific, Inc.) and double distilled water. Subsequently, the samples were boiled at 95°C for 5 min and separated by SDS-PAGE using 10 and 12% gels for occludin and claudins, respectively. Proteins were transferred to PVDF membranes (cat. no. IPVH00010, EMD Millipore) and blocked with 5% milk in TBS-Tween 20 (TBS-T) for 2 h at room temperature. Membranes were incubated with constant agitation for 1 h at room temperature with the following primary antibodies (all from Santa Cruz Biotechnology, Inc.) diluted with 1% milk in TBS-T: Rabbit anti-occludin (1:500; cat. no. sc-5562); rabbit anti-claudin-2 (1:500; cat. no. sc-133464); goat anti-claudin-4 (1:1,000; cat. no. sc-17664); goat anti-claudin-7 (1:1,000; cat. no. sc-17670); rabbit anti-claudin-12 (1:500; cat. no. sc-98608); rabbit anti-claudin-15 (1:500; cat. no. sc-25712) and actin (1:1,000; cat. no. sc-1615). The membranes were subsequently incubated with continuous agitation for 1 h at room temperature with HRP-conjugated goat anti-rabbit IgG (cat. no. 31460, Invitrogen; Thermo Fisher Scientific, Inc.) or HRP-conjugated rabbit anti-goat IgG (1:5,000; cat. no. 81-1620, Invitrogen; Thermo Fisher Scientific, Inc.) diluted with 1% milk in TBS-T. Finally, the blots were developed using a SuperSignal West Femto enhanced chemiluminescence kit (cat. no. 34096; Thermo Fisher Scientific, Inc.), and images were captured using a Fusion SL system (Vilber Lourmat). Protein expression was quantified using VisionCape Advance version 16.11a software (Vilber Lourmat).

#### Counting of colonic aerobic bacteria

After the samples were weighed, 1-cm colonic segments collected in sterile microcentrifuge tubes (2 ml) containing thioglycolate broth (500 *µl*) were fully homogenized. Colonic suspensions were serially diluted (×10) in thioglycolate broth, and 10 *µl* of the serial dilutions were plated on trypticase soy agar. Following incubation for 48 h at 37°C, the number of colonies was counted to determine the colony-forming units (CFU)/g.

#### Statistical analysis

Experimental assays were repeated three times (n=36), and representative data from one assay (n=12) are presented. Data are expressed as the mean ± standard deviation (n=6/group) and were compared using parametric (Student's t-test) or nonparametric (Mann-Whitney test) tests. All data were analyzed using SigmaPlot for Windows version 11.1 (Systat Software Inc.). P<0.05 was considered to indicate a statistically significant difference.

## Results

### 

#### Stress decreases TJ mRNA and protein expression in the colon

**Figure 1.**
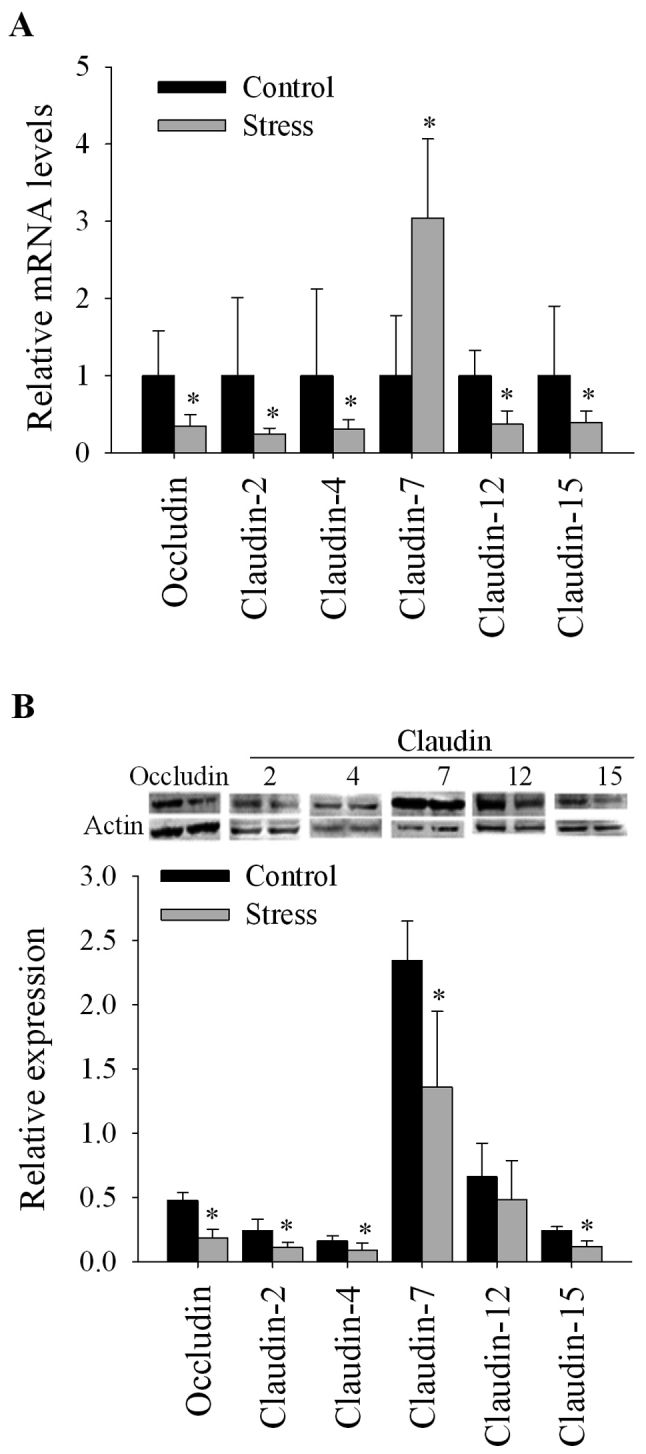
Expression of tight junction proteins in the colon of immobilization-stressed mice. Relative (A) mRNA and (B) protein expression of occludin and claudin-2, −4, −7, −12 and −15 in the colon of mice subject to chronic immobilization stress or unstressed control mice. n=6/group. Data are presented as the mean ± standard deviation. *P<0.05 vs. control.

Stress affects the barrier function of the epithelial cell layer by modulating TJ protein expression (16–19); therefore, transcriptional and translational expression of TJ proteins was determined. At the transcriptional level, mice subjected to short-term repeated immobilization stress exhibited significantly reduced mRNA expression of occludin, and claudin −2, −4, −12 and −15 (P<0.05) and increased mRNA levels of claudin-7 (P<0.05) compared with the control group (Fig. 1). At the protein level, stressed mice exhibited significantly reduced expression of occludin, and claudin −2, −4, −7 and −15 (P<0.05) compared with control animals. Additionally, the reduced claudin-12 expression in the stressed group was not statistically significant (Fig. 1).

#### Chronic immobilization stress has no effect on colonic inflammatory cell responses, but alters goblet cell cellularity

**Figure 2.**
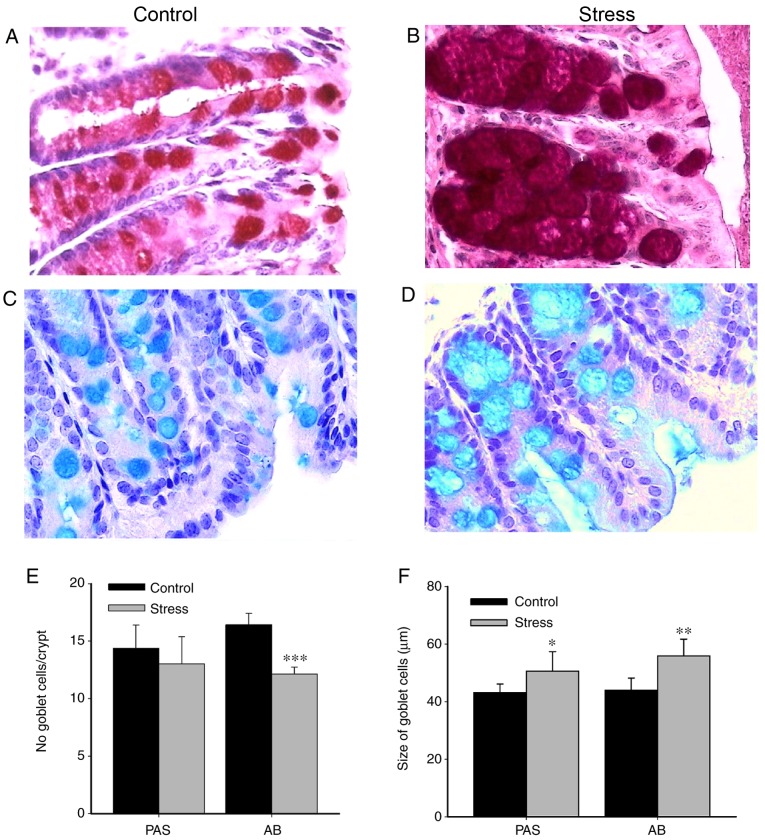
Morphological analysis of the colons of immobilization-stressed mice. Representative images of colonic goblet cells in control and mice subjected to chronic immobilization stress (magnification, ×40). Neutral mucins were identified by PAS staining of (A) control and (B) mice subjected to chronic immobilization stress. Acid mucins were identified by AB staining of (C) control and (D) mice subjected to chronic immobilization stress. (E) Number of goblet cells/crypt and (F) size of goblet cells in colon samples from control and stressed mice, as determined by PAS and AB staining. n=6/group. Data are presented as the mean ± standard deviation. *P<0.05, **P<0.01 and ***P<0.001 vs. control. PAS, periodic acid-Schiff; AB, alcian blue.

Chronic stress impairs gut barrier function by increasing the luminal infiltration of proinflammatory cells (9,11) and altering intestinal mucosal cells (28). Therefore, the effect of immobilization stress on the colonic proinflammatory response was investigated (Fig. 2). Compared with the control unstressed group, the colonic infiltration of polymorphonuclear leukocytes was not significantly altered in the stressed group (data not shown). Histological analysis was performed to identify and evaluate the presence of goblet cells, which are the major producers of mucins. AB and PAS staining were conducted to detect acid and neutral mucopolysaccharides, respectively. Compared with the control, goblet cells in the colons of stressed animals exhibited markedly increased granule density of neutral (Fig. 2A and B) and acid mucins (Fig. 2C and D). Furthermore, the number of PAS-reactive goblet cells was not significantly different between the control and stressed groups; however, the number of AB-reactive goblet cells was significantly decreased in colonic samples from stressed mice stained compared with the control (P<0.001; Fig. 2E). Conversely, the size of goblet cells was significantly increased in the stressed group compared with the control (PAS, P<0.05; AB, P<0.01; Fig. 2F).

#### Stress elicits an increase in fecal lactoferrin levels

**Figure 3.**
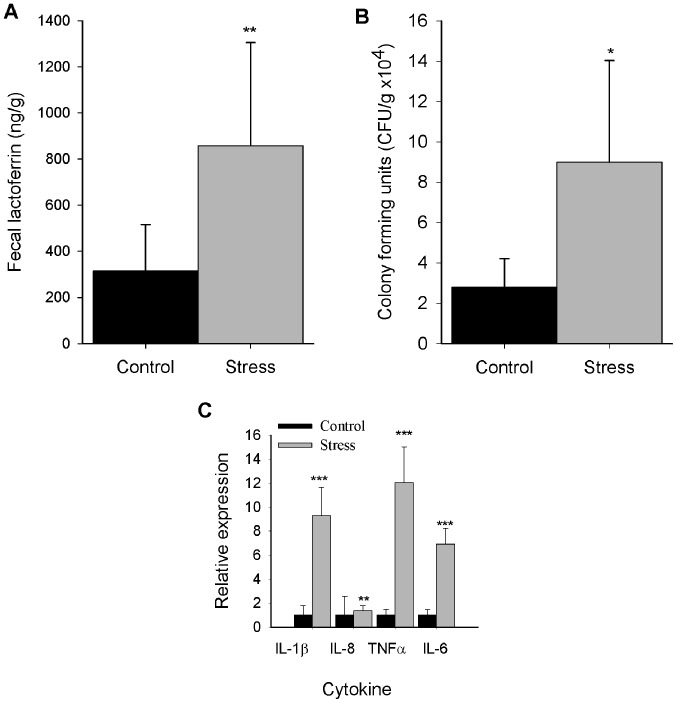
Expression of proinflammatory biomarkers in the colon of immobilization-stressed mice. (A) Fecal lactoferrin (ng/g). (B) CFU/g of colonic aerobic bacteria. (C) Relative mRNA expression of IL-1β, IL-8, TNF-α and IL-6 in colonic mucosa. n=6/group. Data are presented as the mean ± standard deviation. *P<0.05, **P<0.01 and ***P<0.001 vs. control. CFU, colony-forming units; IL, interleukin; TNF-α, tumor necrosis factor-α.

Fecal lactoferrin is a noncellular biomarker of inflammation that serves an important role in gut barrier function (23,29). Thus, fecal lactoferrin was quantified to investigate the effects of repeated immobilization stress on inflammation. As presented in Fig. 3A, fecal extracts from the stressed group exhibited significantly increased levels of fecal lactoferrin compared with the control group (P<0.01).

#### Stress induces colonic growth of aerobic bacteria

Stress induces deleterious effects on intestinal homeostasis by promoting the overgrowth of microbiota members, leading to dysbiosis (30); therefore, the abundance of aerobic bacteria was determined in the stress model of the present study. Compared with the control, a significant increase in aerobic bacterial colony formation was detected in the colons of stressed mice (P<0.05; Fig. 3B).

#### Stress induces mRNA expression of proinflammatory cytokines

As previously reported, chronic stress alters TJ protein expression by eliciting the generation of components involved in the inflammatory response, such as tumor necrosis factor (TNF)-α and interleukin (IL)-1β (17). Therefore, the expression of inflammatory cytokines in colonic mucosa was determined. It was revealed that chronic stress upregulated the expression TNF-α, IL-1β, IL-6 (P<0.001) and IL-8 (P<0.01) mRNA levels compared with the control group (Fig. 3C).

## Discussion

The effects of chronic stress on transcriptional and/or protein expression of TJ proteins has been analyzed in rats and mice subjected to water-avoidance stress and crowding stress (16–19); however, to the best of our knowledge, the present study is the first report of the effects of chronic immobilization stress on the colonic expression of occludin, and claudin-2, −4, −7, −12 and −15. It was observed that immobilization stress downregulated the expression of TJ proteins at the transcriptional and protein levels.

As previously reported following water-avoidance stress in mice and rats, stress reduced the transcription and protein expression of occludin (16–19). Occludin collaborates with claudin-2 in the regulation of the paracellular flux of solutes and ultimately barrier function (3). Occludin serves a role in promoting a leaky pathway in the epithelium suggesting unrestricted macromolecule flux; however, claudin-2 is a pore-forming claudin involved in the restricted diffusion of ions via the pore pathway (31). It has been hypothesized that interactions between dephosphorylated or phosphorylated forms of occludin and claudin-2 via ZO-1 control the regulation of leaky or pore-forming TJ pathways, respectively (3).

Previous studies using water-avoidance stress or crowding stress on rats revealed that claudin-2 protein expression was unchanged or increased, and positively associated with TNF-α mRNA levels following stress (17,18). In the present study, repeated immobilization stress downregulated claudin-2 expression at the transcriptional and protein level, whereas stress induced an increase in the mRNA expression of TNF-α and other proinflammatory cytokines, including IL-1β and IL-6. The discrepancy may reflect independent TNF-α mechanisms underlying claudin-2 expression during stress responses that may involve mediators of oxidative burst, such as reactive oxygen species (ROS) (11,32). It was previously reported that ROS, such as hydrogen peroxide, decreased the expression of claudin-2 and occludin in polarized Caco-2 cell monolayer cultures (33).

In the present study, immobilization stress downregulated claudin-4, a pore-sealing claudin, and claudin-7, −12 and −15, which are regarded as pore-forming claudins that permit cation flux (4,34). Claudin-7 exhibited either increased mRNA expression or reduced protein expression in stressed animals; this discrepancy potentially resulted from decreased translation of claudin-7 mRNA or rapid protein turnover (6). Divergence between the significant decrease in claudin-12 mRNA and the non-significant effect on claudin-12 protein (potentially due to data dispersion) was observed. Despite the mismatch, the findings suggested an inhibitory effect of stress on claudin-12 expression. In the present study, claudin-7 was overexpressed; however, the underlying mechanism remains unclear. Dissimilar to other claudins, claudin-7 is distributed and highly expressed evenly throughout the intestinal tract, and located more prominently on the basolateral membrane compared with the apical tips of epithelial cells (4).

Experimental data from male rats or male BALB/c mice indicated that chronic water-avoidance stress evoked a mast cell-dependent response associated with mucus depletion, concomitant inflammation as determined by increased infiltration of mononuclear cells and/or neutrophils, and a reduced ratio of colonic goblet cells to epithelial cells (9,11). In other studies, histological differences (morphology and inflammation) in the colons of male mice under restraint stress and control mice were not observed (35). In the present study, chronic stress reduced the goblet cell number and induced the enlargement of their size and granule density, as determined via AB staining. The stress-induced reduction in goblet cell number may induce proinflammatory effects by decreasing the mucus layer thickness, increasing contact between luminal bacteria and the epithelial surface (36). Furthermore, in the current study, chronic stress elicited the transcription of IL-8, a potent chemokine; however, a significant increase in polymorphonuclear leukocyte infiltration was not observed (data not shown). As the experiments presented in this study were conducted in female mice, inconsistencies between the present and previous findings may arise, in part, due to the effects of sexual dimorphism of colonic homeostasis (37). Compared with male and postmenopausal counterparts, young fertile females exhibit more robust intestinal homeostasis and are less prone to the effects of chronic stress on the inflammatory response, due in part to the protective role of estrogens (38).

According to the present findings, immobilization stress elicited the production of fecal lactoferrin, which is regarded as an inflammatory biomarker. To the best of our knowledge, the effects of immobilization stress on colonic inflammation as determined by fecal lactoferrin have not been previously reported. As previously demonstrated, the induction of fecal lactoferrin production is caused by increased luminal infiltration of circulant neutrophils, as observed in inflammatory conditions, such as those induced by gut infections and intestinal bowel disease (23,29). In this assay, increased production of fecal lactoferrin and IL-8 mRNA (a neutrophil-chemotactic chemokine) (39) was observed; however, no significant differences in luminal infiltration of neutrophils were detected (data not shown). The latter finding may suggest an inhibitory effect of the stress glucocorticoid response on colonic neutrophil recruitment and/or proliferation, as described in models of colitis, including water-avoidance stress and dinitrofluorobenzene (40,41). Thus, the stimulation of fecal lactoferrin production suggests an increased transudation of circulant lactoferrin released by degranulated neutrophils (29), demonstrating the complex interplay of neuroendocrine and immune pathways that regulate the gut barrier function, as observed in intestinal diseases associated with an emotional component, such as irritable bowel syndrome (42). In this study, the overgrowth of aerobic bacteria was observed. It was previously reported that, under stress conditions, catecholamine stress hormones separate iron from iron-binding proteins, such as lactoferrin, promoting iron uptake and eliciting intestinal bacteria growth (30).

In the present study, the permeability of the epithelial barrier was not determined; however, the function of the epithelial barrier can be unaffected, despite an increase in neutrophil transmigration induced by occludin disruption (43). Additionally, the expression of proinflammatory cytokines was determined exclusively via RT-qPCR analysis without conducting functional assays (ELISA and/or flow cytometry). Even with these limitations, the animal study provides an experimental background to inform research intended for the development of therapies to treat dysfunctions in which stress has a prominent proinflammatory role, such as inflammatory bowel syndrome, and chronic inflammatory diseases in which stress is a contributing factor, such as ulcerative colitis (44,45).

In conclusion, the findings from the present study revealed that short-term immobilization stress downregulated the expression of TJ proteins, and induced a proinflammatory response involving the production of fecal lactoferrin, the expression of proinflammatory cytokines at the mRNA level and an overgrowth of gut aerobic bacteria. The study provides novel insight regarding the expression of TJ proteins in the intestinal epithelium and inflammation in response to short-term immobilization stress. These findings may reflect underlying mechanisms involving crosstalk between components of the systemic and local neuroendocrine responses aimed at reestablishing colonic homeostasis in the gut barrier during and following chronic stress.

## Figures and Tables

**Table I.** Primer set used for reverse transcription-quantitative PCR analysis of tight junction proteins and cytokines.

Gene	Forward primer (5′-3′)	Reverse primer (5′-3′)
β-actin	ATTGGCAATGAGCGGTTCA	GGATGCCACAGGACTCCAT
Occludin	ATGTCCGGCCGATGCTCTC	CTTTGGCTGCTGTTGGGTCTG
Claudin-2	GGCTGTTAGGCACATCCAT	TGGCACCAACATAGGAACTC
Claudin-4	ACAGGTCCTGGGAATCTCCT	CACTGCATCTGACCTGTCCT
Claudin-7	CACACGCCTTTAATCCCAGT	TGATGTCTCCCAAGTCCACA
Claudin-12	AAGTGGCCGAGGAGGTATTT	GAGCAGGTCCGCGTTACACA
Claudin-15	TGAGGCTTGGCTGTTTCTTT	AAGCCTGGCAGCTTAAAACA
IL-6	CCCCAATTTCCAATGCTCTCC	CGCACTAGGTTTGCCGAGTA
IL-8	TGCATGGACAGTCATCCCC	ATGACAGACCACAGAACGGC
IL-1β	TGCCACCTTTTGACAGTGATG	TGATGTGCTGCTGCGAGATT
TNF-α	GATCGGTCCCCAAAGGGATG	TTTGCTACGACGTGGGCTAC

IL, interleukin; TNF-α, tumor necrosis factor-α.

## Data Availability

All data generated or analyzed during the present study are included in this published article.

## References

[1] Zhang K, Hornef MW, Dupont A (2015). The intestinal epithelium as guardian of gut barrier integrity. Cell Microbiol.

[2] France MM, Turner JR (2017). The mucosal barrier at a glance. J Cell Sci.

[3] Turner JR, Buschmann MM, Romero-Calvo I, Sailer A, Shen L (2014). The role of molecular remodeling in differential regulation of tight junction permeability. Semin Cell Dev Biol.

[4] Fujita H, Chiba H, Yokozaki H, Sakai N, Sugimoto K, Wada T, Kojima T, Yamashita T, Sawada N (2006). Differential expression and subcellular localization of claudin-7, −8, −12, −13, and −15 along the mouse intestine. J Histochem Cytochem.

[5] Holmes JL, Van Itallie CM, Rasmussen JE, Anderson JM (2006). Claudin profiling in the mouse during postnatal intestinal development and along the gastrointestinal tract reveals complex expression patterns. Gene Expr Patterns.

[6] Inai T, Sengoku A, Guan X, Hirose E, Iida H, Shibata Y (2005). Heterogeneity in expression and subcellular localization of tight junction proteins, claudin-10 and −15, examined by RT-PCR and immunofluorescence microscopy. Arch Histol Cytol.

[7] Faderl M, Noti M, Corazza N, Mueller C (2015). Keeping bugs in check: The mucus layer as a critical component in maintaining intestinal homeostasis. IUBMB Life.

[8] Bailey MT, Dowd SE, Parry NM, Galley JD, Schauer DB, Lyte M (2010). Stressor exposure disrupts commensal microbial populations in the intestines and leads to increased colonization by Citrobacter rodentium. Infect Immun.

[9] Cameron HL, Perdue MH (2005). Stress impairs murine intestinal barrier function: Improvement by glucagon-like peptide-2. J Pharmacol Exp Ther.

[10] Reber SO, Peters S, Slattery DA, Hofmann C, Schölmerich J, Neumann ID, Obermeier F (2011). Mucosal immunosuppression and epithelial barrier defects are key events in murine psychosocial stress-induced colitis. Brain Behav Immun.

[11] Söderholm JD, Yang PC, Ceponis P, Vohra A, Riddell R, Sherman PM, Perdue MH (2002). Chronic stress induces mast cell-dependent bacterial adherence and initiates mucosal inflammation in rat intestine. Gastroenterology.

[12] Santos J, Yang PC, Söderholm JD, Benjamin M, Perdue MH (2001). Role of mast cells in chronic stress induced colonic epithelial barrier dysfunction in the rat. Gut.

[13] Santos J, Yates D, Guilarte M, Vicario M, Alonso C, Perdue MH (2008). Stress neuropeptides evoke epithelial responses via mast cell activation in the rat colon. Psychoneuroendocrinology.

[14] Vicario M, Guilarte M, Alonso C, Yang P, Martínez C, Ramos L, Lobo B, González A, Guilà M, Pigrau M (2010). Chronological assessment of mast cell-mediated gut dysfunction and mucosal inflammation in a rat model of chronic psychosocial stress. Brain Behav Immun.

[15] Vicario M, Alonso C, Guilarte M, Serra J, Martínez C, González-Castro AM, Lobo B, Antolín M, Andreu AL, García-Arumí E (2012). Chronic psychosocial stress induces reversible mitochondrial damage and corticotropin-releasing factor receptor type-1 upregulation in the rat intestine and IBS-like gut dysfunction. Psychoneuroendocrinology.

[16] Zheng G, Wu SP, Hu Y, Smith DE, Wiley JW, Hong S (2013). Corticosterone mediates stress-related increased intestinal permeability in a region-specific manner. Neurogastroenterol Motil.

[17] Hattay P, Prusator DK, Tran L, Greenwood-Van Meerveld B (2017). Psychological stress-induced colonic barrier dysfunction: Role of immune-mediated mechanisms. Neurogastroenterol Motil.

[18] Lauffer A, Vanuytsel T, Vanormelingen C, Vanheel H, Salim Rasoel S, Tóth J, Tack J, Fornari F, Farré R (2016). Subacute stress and chronic stress interact to decrease intestinal barrier function in rats. Stress.

[19] Nébot-Vivinus M, Harkat C, Bzioueche H, Cartier C, Plichon-Dainese R, Moussa L, Eutamene H, Pishvaie D, Holowacz S, Seyrig C (2014). Multispecies probiotic protects gut barrier function in experimental models. World J Gastroenterol.

[20] Official Mexican Standard NOM-062-ZOO-1999 (2001). Technical specifications for the production, care and use of laboratory animals. Secretary of agriculture, livestock, rural development, fisheries and Food (SAGARPA). Official Gazette, Mexican Federal Government.

[21] Bhatia N, Maiti PP, Choudhary A, Tuli A, Masih D, Khan U, Ara T, Jaggi AS (2011). Animal models in the study of stress: A review. NSHM J Pharm Healthcare Manage.

[22] Uni Z, Smirnov A, Sklan D (2003). Pre- and posthatch development of goblet cells in the broiler small intestine: Effect of delayed access to feed. Poult Sci.

[23] Logsdon LK, Mecsas J (2006). A non-invasive quantitative assay to measure murine intestinal inflammation using the neutrophil marker lactoferrin. J Immunol Methods.

[24] Xiao Y, Isaacs SN (2012). Enzyme-linked immunosorbent assay (ELISA) and blocking with bovine serum albumin (BSA)-not all BSAs are alike. J Immunol Methods.

[25] Livak KJ, Schmittgen TD (2001). Analysis of relative gene expression data using real-time quantitative PCR and the 2(-Delta Delta C(T)) method. Methods.

[26] Rozen S, Skaletsky H (2000). Primer3 on the WWW for general users and for biologist programmers. Methods Mol Biol.

[27] Kyoko OO, Kono H, Ishimaru K, Miyake K, Kubota T, Ogawa H, Okumura K, Shibata S, Nakao A (2014). Expressions of tight junction proteins Occludin and Claudin-1 are under the circadian control in the mouse large intestine: Implications in intestinal permeability and susceptibility to colitis. PLoS One.

[28] Liévin-L Moal V, Servin AL (2006). The front line of enteric host defense against unwelcome intrusion of harmful microorganisms: Mucins, antimicrobial peptides, and microbiota. Clin Microbiol Rev.

[29] Siddiqui I, Majid H, Abid S (2017). Update on clinical and research application of fecal biomarkers for gastrointestinal diseases. World J Gastrointest Pharmacol Ther.

[30] Sandrini SM, Shergill R, Woodward J, Muralikuttan R, Haigh RD, Lyte M, Freestone PP (2010). Elucidation of the mechanism by which catecholamine stress hormones liberate iron from the innate immune defense proteins transferrin and lactoferrin. J Bacteriol.

[31] Al-Sadi R, Khatib K, Guo S, Ye D, Youssef M, Ma T (2011). Occludin regulates macromolecule flux across the intestinal epithelial tight junction barrier. Am J Physiol Gastrointest Liver Physiol.

[32] Ponferrada A, Caso JR, Alou L, Colón A, Sevillano D, Moro MA, Lizasoain I, Menchén P, Gómez-Lus ML, Lorenzo P (2007). The role of PPARgamma on restoration of colonic homeostasis after experimental stress-induced inflammation and dysfunction. Gastroenterology.

[33] Iraha A, Chinen H, Hokama A, Yonashiro T, Kinjo T, Kishimoto K, Nakamoto M, Hirata T, Kinjo N, Higa F (2013). Fucoidan enhances intestinal barrier function by upregulating the expression of claudin-1. World J Gastroenterol.

[34] Nevado R, Forcén R, Layunta E, Murillo MD, Grasa L (2015). Neomycin and bacitracin reduce the intestinal permeability in mice and increase the expression of some tight-junction proteins. Rev Esp Enferm Dig.

[35] Koh SJ, Kim JW, Kim BG, Lee KL, Kim JS (2015). Restraint stress induces and exacerbates intestinal inflammation in interleukin-10 deficient mice. World J Gastroenterol.

[36] Kim JJ, Khan WI (2013). Goblet cells and mucins: Role in innate defense in enteric infections. Pathogens.

[37] Bourke CH, Harrell CS, Neigh GN (2012). Stress-induced sex differences: Adaptations mediated by the glucocorticoid receptor. Horm Behav.

[38] Grishina I, Fenton A, Sankaran-Walters S (2014). Gender differences, aging and hormonal status in mucosal injury and repair. Aging Dis.

[39] Andrews C, McLean MH, Durum SK (2018). Cytokine tuning of intestinal epithelial function. Front Immunol.

[40] Cakir B, Bozkurt A, Ercan F, Yeğen BC (2004). The anti-inflammatory effect of leptin on experimental colitis: Involvement of endogenous glucocorticoids. Peptides.

[41] Rijnierse A, Koster AS, Nijkamp FP, Kraneveld AD (2006). TNF-alpha is crucial for the development of mast cell-dependent colitis in mice. Am J Physiol Gastrointest Liver Physiol.

[42] Camilleri M, Sellin JH, Barrett KE (2017). Pathophysiology, evaluation, and management of chronic watery diarrhea. Gastroenterology.

[43] Lapointe TK, Buret AG (2012). Interleukin-18 facilitates neutrophil transmigration via myosin light chain kinase-dependent disruption of occludin, without altering epithelial permeability. Am J Physiol Gastrointest Liver Physiol.

[44] Ananthakrishnan AN (2013). Environmental triggers for inflammatory bowel disease. Curr Gastroenterol Rep.

[45] Konturek PC, Brzozowski T, Konturek SJ (2011). Stress and the gut: Pathophysiology, clinical consequences, diagnostic approach and treatment options. J Physiol Pharmacol.

